# Skeletal Muscle Laminopathies: A Review of Clinical and Molecular Features

**DOI:** 10.3390/cells5030033

**Published:** 2016-08-11

**Authors:** Lorenzo Maggi, Nicola Carboni, Pia Bernasconi

**Affiliations:** 1 Neurology IV—Neuroimmunology and Neuromuscular Diseases Unit, Fondazione Istituto Neurologico Carlo Besta, 20133 Milan, Italy; pia.bernasconi@istituto-besta.it; 2 Neurology Department, Hospital San Francesco of Nuoro, 08100 Nuoro, Italy; nikola.carboni@tiscali.it

**Keywords:** laminopathies, *LMNA* gene, cardiomyopathy, arrhythmia, limb-girdle muscular dystrophy, congenital muscular dystrophy, Emery-Dreifuss muscular dystrophy

## Abstract

*LMNA*-related disorders are caused by mutations in the *LMNA* gene, which encodes for the nuclear envelope proteins, lamin A and C, via alternative splicing. Laminopathies are associated with a wide range of disease phenotypes, including neuromuscular, cardiac, metabolic disorders and premature aging syndromes. The most frequent diseases associated with mutations in the *LMNA* gene are characterized by skeletal and cardiac muscle involvement. This review will focus on genetics and clinical features of laminopathies affecting primarily skeletal muscle. Although only symptomatic treatment is available for these patients, many achievements have been made in clarifying the pathogenesis and improving the management of these diseases.

## 1. Introduction

*LMNA*-related disorders are rare diseases caused by mutations in the *LMNA* gene, which encodes for the nuclear envelope proteins, lamin A and C, via alternative splicing [1]. These proteins play a role in several cellular processes, and mutations in the *LMNA* gene are associated with a wide range of disease phenotypes, ranging from neuromuscular, cardiac and metabolic disorders to premature aging syndromes [2,3,4,5,6].

The most frequent diseases associated with *LMNA* mutations are characterized by skeletal and cardiac muscle involvement. Different phenotypes have been reported with skeletal muscle involvement: limb-girdle muscular dystrophy type 1B [LGMD1B, Online Mendelian Inheritance in Man (OMIM) *150330 #159001 or *LMNA*-related dystrophy (*LMNA*-RD)]; autosomal dominant Emery-Dreifuss muscular dystrophy (EDMD2, OMIM#181350 or *LMNA*-RD); and a form of congenital muscular dystrophy (*LMNA*-CMD, OMIM#613205 or *LMNA*-RD) [7]. Heart involvement shows a high penetrance, and almost all patients after the seventh decade of life show cardiac diseases, regardless of the presence of a myopathy. On the contrary, dilated cardiomyopathy may exist without muscle disease, although subtle muscle involvement may be present and underestimated.

This review will focus on the genetics and clinical features of laminopathies affecting primarily skeletal muscle.

## 2. Genetics and Pathogenesis

Lamins constitute a network of intermediate filament proteins underlying the inner nuclear membrane, which constitutes the nuclear envelope together with the outer nuclear membrane [8]. The major components of this network are two lamin subgroups—A-type (lamins A and C) and B-type lamins (lamins B1 and B2)—and the associated proteins (more than 100, such as nesprin, actin, the nuclear prelamin A recognition factor or NARF, the Sad1 and UNC84 domain-containing protein 1 or SUN1) [8,9,10,11,12]. The lamins, in particular the A-type lamins, stabilize mechanically the nucleus [13,14], are involved in ‘chromatin dynamics’ [15,16] and influence a myriad of signaling pathways [9,17,18]. A-type lamins are mainly expressed in differentiated cells, whereas B-type lamins are expressed by all cells throughout development [19]. A-type lamins are produced by alternative splicing of a single gene, the *LMNA* gene, which also encodes lamin C2 and lamin A delta 10 [19,20]. B-type lamins and lamin A are expressed as precursor proteins and undergo several steps of post-translational modifications (cysteine farnesylation and carboxymethylation) at the C-terminal CaaX box domain. Prelamin A is further processed by the zinc metalloprotease related to Ste24p (Zmpste24/FACE1), which originates the lamin A mature form devoid of the 15 C-terminal amino acids [21].

Although lamins A/C are expressed in almost all cells and tissues, the high degree of tissue-specificity (i.e., skeletal muscle, cardiac muscle, adipose tissue, peripheral nerve) and the resulting different phenotypes observed in laminopathies are not completely elucidated [22]. For this purpose, a predilection for skeletal and cardiac muscle is remarkable, accounting for about 80% of total *LMNA* mutations [2]. All types of mutation have been found in laminopathies, including missense, nonsense, in-frame and out-of-frame insertions/deletions, splice site mutations and, rarely, large exonic deletions. Among them, missense mutations are by far the most frequent type of mutations observed in laminopathies, including skeletal muscle laminopathies [2,5]. Two hypotheses, not mutually exclusive, have been formulated to explain why specific cells, and in particular muscle and cardiac cells, are more sensitive to A-type lamin expression alterations: the ‘structural’ hypothesis and the ‘gene regulation’ hypothesis [2,3,22].

The ‘structural’ hypothesis suggests that mutated A-type lamins or the associated nuclear envelope proteins disrupt the integrity of the cell nuclear membrane, resulting in nuclear breakage and cell death in tissues exposed to mechanical stress, such as muscle fibers [3,22,23]. Furthermore, lamins play a prominent role in nucleo-cytoskeletal coupling by the interactions with components of LINC (linker of nucleoskeleton to cytoskeleton complex). This interaction is fundamental for several cellular functions, including positioning of synaptic nuclei in muscle fibers. It is likely that *LMNA* mutations alter the interaction between the nucleus and the cytoskeleton, perturbing the intracellular force transmission and preventing retrograde nuclear movement of proteins [14,23,24].

The ‘gene regulation hypothesis’ suggests that A-type lamins are crucial in tissue-specific gene expression. Indeed, in EDMD muscle, the transcriptional regulation is defective, likely due to a focal loss and disorganization of heterochromatin in fibroblast and muscle fiber nuclei (reviewed in [16]). Lamins are known to interact with transcriptional regulators, such as c-Fos, ERK1/2, pRB and SREBP1 [23,25]. Mutations in the *LMNA* gene might alter the intranuclear localization of these factors and their stability, perturbing the activation of important signaling pathways, such as mitogen-activated protein kinases (MAPK), ERK, JNK, p38α and mTOR and, hence, negatively affecting the transcription of the factor-regulated genes. Interestingly, identification of the altered signaling pathway might represent a suitable target for a therapeutic intervention: treatments with rapamycin or MAPK inhibitors have been shown to improve symptoms in EDMD and dilated cardiomyopathy animal models [18,25,26,27,28].

Growing evidences suggest that a ‘quality control’ mechanism, which preserves nuclear pore complex function and nuclear envelop integrity, ensuring the correct nuclear compartmentalization, might occur also in mammalian cells [29]. It will be of interest to understand how this mechanism acts in laminopathy cells. Indeed, it has been demonstrated that induction of the autophagic process in mutant cells counteracts the negative effect of H222P *LMNA* mutation on cardiomyopathy in animal models [30].

Besides the above-mentioned hypotheses, it is becoming clear that, even if nuclear lamins and nuclear envelope transmembrane proteins are widely expressed, the composition of the nuclear envelope can vary according to the cell type; this tissue-specific ‘specialized’ structure might account for the variability observed in laminopathies [31].

## 3. Skeletal Muscle Involvement in Laminopathies

*LMNA*-related myopathies (*LMNA*-RD) represent a consistent subgroup among diseases due to mutations in the *LMNA* gene. Three main myopathic phenotypes have been reported based on the distribution of muscle weakness or age at onset: LGMD1B, EDMD2 and *LMNA*-CMD (Table 1). However, these clinical entities may be determined by the same *LMNA* mutation and coexist in the context of the same family [5,32,33]; furthermore, due to the considerable clinical overlap, these phenotypes should be considered as a continuum in the clinical spectrum of *LMNA*-RD. Indeed, heart is affected in all three entities, with similar features, except for younger age at onset in *LMNA*-CMD and EDMD2 than LGMD1B, which also has later presentation of muscle weakness [5]. Of note, cardiac presentation may precede the onset of muscle weakness.

Creatine kinase is usually normal or mildly elevated (<5-times upper normal value) in *LMNA*-RD. Electromyography is usually not relevant for diagnosis, showing aspecific myopathic patterns. Histological findings in the muscle tissue are usually unspecific, sometimes displaying dystrophic features, making muscle biopsy not necessary for diagnosis in patients with typical clinical features. Western blot for lamin A/C [34] showed reduced protein levels only in about half of the patients, and considering also the required technical expertise, its role in the diagnosis workup of skeletal muscle laminopathies is substantially not relevant. Muscle MRI may be helpful in differential diagnosis towards other myopathies, *LMNA*-RD being associated with predominant fatty infiltration of medial gastrocnemius and vasti muscles with relative sparing of the rectus femoris [35,36]. Whole body MRI in pediatric *LMNA*-RD displayed predominant signal abnormalities in erector spinae, serratus anterior, subscapularis, gluteus medius and minimus, vasti adductor magnus and longus, semimembranosus, medial gastrocnemius and soleus muscles [37].

**Figure 1:**
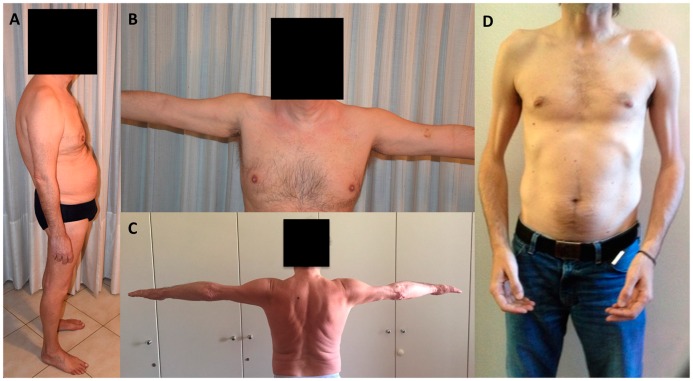
Description of typical clinical features of *LMNA*-related dystrophy (*LMNA*-RD). Patient affected by LGMD1B phenotype: note the lumbar lordosis (**A**) and upper limb abduction weakness (**B**); patient with LGMD1B: note the humeral atrophy and absence of scapular winging, which is typically associated with EDMD2 (**C**); patient affected by EDMD2 phenotype: note the elbow flexion contractures and wasting of humeral muscles (**D**).

EDMD2 was the first described myopathic phenotype [38] and is clinically characterized by the triad of early ankle, elbow and spine contractures, muscle wasting and weakness in a scapulo-humero-peroneal distribution, in particular in early disease stages (see Figure 1), and heart involvement presenting in adult life with dilated cardiomyopathy and conduction system defects associated with high risk of cardiac sudden death [39,40]. Muscle weakness usually occurs within the beginning of the second decade [5,41], and it is sometimes preceded by contractures, which may be severe and impair posture and gait. Compared to EDMD2, the X-linked Emery-Dreifuss muscular dystrophy (EDMD1) caused by mutations in the *EMD* gene, displays a humero-peroneal distribution of weakness, contractures as the presenting symptoms, less common loss of walking ability and lower risk of sustained ventricular tachyarrhythmia and dilated cardiomyopathy [42,43,44]. A recent study showed a similar pattern of fatty infiltration in myopathic patients with mutations in the *EMD* and *LMNA* gene, with the main muscles involved being the paravertebral, glutei, vasti, biceps, semitendinosus, semimembranosus, adductor major, soleus and gastrocnemius; however, involvement of peroneus muscle pointed to *EMD* gene mutations [45]. Few cases of X-linked EDMD due to mutations in the *FHL1* gene have been described, differing from EDMD1 and EDMD2 for the detection of hypertrophic cardiomyopathy [46].

LGMD1B differs from EDMD2 by the distribution of muscle wasting and weakness, being characterized by predominant scapular and pelvic girdle muscle involvement. However, in later stages of the disease, differential diagnosis between EDMD2 and LGMD1B may be challenging due to pelvic muscle weakness developing also in the former phenotype. Furthermore, LGMD1B age at onset is later than EDMD2, usually in the third or fourth decades. Of note, contractures, which were initially considered absent or late in disease course, have been recently found in about two-thirds of LGMD1B patients, sometimes in early disease stages, although elbow contractures should be considered more specific for EDMD2 [5,47].

More recently, a form of *LMNA*-CMD has been described in patients presenting at birth or within the first two years of life [34,48]; in particular, two phenotypes have been observed: a severe congenital form with minimal or absent motor development and a milder and more frequent myopathy characterized by prominent axial weakness defined as dropped head syndrome, after normal acquisition of head control, usually with preservation of walking ability [5,48]. Rigid spine and scoliosis are relatively frequent [5]; contractures are almost invariably detected, presenting initially in distal limbs and then developing in proximal joints [5,48]. *LMNA*-CMD patients may progress both to EDMD2 or LGMD1B [5,48]. Contrary to EDMD2 and LGMD1B, respiratory failure is very frequent, whereas cardiac involvement is less common, but this may be related to the young age of the patients when evaluated. Of note, sudden death has been reported also in the first decade of life [48]. Muscle biopsy reveals dystrophic features in more than a half of the patients [48]; inflammatory findings are relatively uncommon [48]. However, *LMNA* mutations were identified in about half of a small cohort of Japanese patients suspected to have an inflammatory myopathy presenting before the age of two years, with some benefit from steroids in four out of the eight treated cases [49]. Central nervous system is not affected in *LMNA*-CMD, except for a single case report on a girl with dropped head syndrome and focal white matter changes without cognitive impairment [50].

A recent study investigating a large cohort of Italian *LMNA*-RD patients demonstrated that LGMD1B was by far the most frequent muscle phenotype and confirmed that the natural history of *LMNA*-RD is mainly marked by heart involvement and related complications [5]. Therefore, cardiologic follow-up is strongly recommended in *LMNA*-RD patients. Progression of muscle weakness was slow over the years, and only six out of 75 patients became wheelchair bound after a mean period of about 20 years from disease onset [5]. Apart from *LMNA*-CMD, respiratory involvement was usually mild, and assisted ventilation was required in a minority of patients [5].

Axonal neuropathy has been reported in a few families carrying the same homozygous *LMNA* mutation (Arg298Cys) and originating from Northwest Africa having Charcot-Marie-Tooth type 2B1 (CMT2B1) [51,52,53]. A family originating from the southwest of France and with mutated *LMNA* gene showed axonal sensorimotor neuropathy in association with muscular dystrophy, cardiac disease and leukonychia [54]. In addition, a small amount of patients (8%), having a myopathy due to *LMNA* mutation, may show unspecific nerve involvement [5], mainly axonal, although it is still not clear if the neuropathy is coincidental or pathologically related to *LMNA* mutations.

## 4. Cardiac Involvement

Mutations on the *LMNA* gene very often are the cause of cardiac compromise, which can occur isolated or be associated with disorders affecting other tissues in the context of laminopathies [55,56]. Usually, the first signs of cardiac compromise are electrical abnormalities, which can include low P wave and prolonged PR interval, with a narrow QRS complex [57]; very often, patients with cardiac compromise *LMNA*-related start showing various rhythm disturbances, which can be unspecific, like sinus bradycardia, sick sinus syndrome, bundle branch block, supraventricular and ventricular ectopic beats, progressive atrioventricular block [58]; although rarely, patients may also develop an atrial paralysis, a severe modality of cardiac compromise typical for cardiolaminopathies [59]. Several studies showed how cardiac compromise seems to be related to age with only a small percentage of patients developing cardiac rhythm abnormalities within the first decade and the majority of patients showing electrical alterations after the third decade [59]. Later on, the modality of cardiac compromise of affected subjects may be further complicated by cardiomyopathy, leading to heart failure [60]; this condition, which occurs less frequently than cardiac rhythm disturbances, is usually preceded by dysrhythmias and atrial tachyarrhythmias [61]. Several studies aimed at reporting the natural history of cardiolaminopathies have shown how patients may die suddenly, even despite the implantation of a device; the reason for this is that subjects with a *LMNA* gene mutation frequently suffer from malignant ventricular tachyarrhythmias, even in the absence of dilated cardiomyopathy [44]. It is not possible to predict the extent and severity of cardiac compromise solely on the basis of the characteristic of the *LMNA* gene mutation; this is confirmed by the marked phenotypic variability among subjects bearing the same lamin A/C gene mutation [59]. As a matter of fact, several authors showed that life style and other variables may modulate the deleterious effect of the mutation, thus helping the clinician in the care of cardiolaminopathies; just as an example, it has been shown that a previous history of competitive sport can produce a deleterious effect of cardiac compromise in subjects bearing a lamin A/C mutation [44]. Furthermore, recent studies have demonstrated how sustained malignant ventricular arrhythmias tend to occur in subjects bearing at least two of the following variables: the status of carrier of a *LMNA* gene mutation other than missense, the male gender, a previous history of non-sustained ventricular arrhythmias and an ejection fraction less than 45% at the first cardiac detection [62].

## 5. Atypical Cases in Laminopathies

The *LMNA*-related atypical cases are clinical entities that do not fit with the diseases classically associated with *LMNA* gene mutations [6]. Atypical forms of laminopathies in the neuromuscular field sharing only part of the classical EDMD2 [63] or LGMD1B or phenotypes placed in between EDMD2/LGMD [35] have also been described. A lethal phenotype with a prenatal onset, dysmorphic facies, limb contractures, fractures of arm and thigh bones, lung hypoplasia and severe generalized muscular dystrophy associated with homozygous nonsense mutation c.777T>A (p.Y259X) has been also reported [64]. Another, unusual, neuromuscular phenotype consists of a clinical entity belonging to the heart-hand syndrome family: the disease was first described in 2005 [65] and recognized as a laminopathy three years later [66]: the authors described a large Slovenian family whose affected members developed from the fourth and fifth decade the typical cardiac compromise that is *LMNA* gene-related, with ECG abnormalities, progressive rhythm conduction defects and late dilated cardiomyopathy. This condition was further complicated by proximal skeletal muscles weakness and brachydactyly mainly affecting the feet, manifesting in its full severity after the sixth decade. This neuromuscular condition is somehow close to non-neuromuscular disorders that are *LMNA* gene-related, including mandibuloacral dysplasia type A (MADA) and progeroid syndromes, in which acroosteolysis leading to shortness of fingers and toes is present. This unique phenotype is caused by an intron 9 mutation (c.1609-12 or IVS9-12T>G) affecting the splicing mechanisms and producing truncating lamins A and C lacking their carboxy terminal domain. Other atypical neuromuscular disorders that are *LMNA*-related include patients with selective hypotrophy and weakness on quadriceps and elbow flexors and mild retractions on elbows, hamstrings or paraspinal muscles, or with mild weakness on neck flexors and foot extensors, or also subjects with severe dilated cardiomyopathy and selective quadriceps myopathy [5].

Of note, atypical phenotypes include also overlapping syndromes obtained by the co-occurrence in the same subject of different diseases caused by the same pathogenic variation on the lamin A/C gene; metabolic alterations in association with skeletal and/or cardiac alterations proved to be the most frequent overlap syndromes: familial partial lipodystrophy, hepatic steatosis or only hypertriglyceridemia can be associated with dilated cardiomyopathy with conduction defects, with or without muscular dystrophy [67]. Other overlapping syndromes may include skeletal and/or cardiac compromise with neuropathy and dermatologic abnormalities, skeletal and/or cardiac compromise, metabolism disturbances and lipodystrophy, MADA/bone alterations variably associated with metabolism abnormalities, premature ageing syndromes, with or without dermatologic abnormalities, and skeletal and cardiac diseases [6].

## 6. Genotype-Phenotype Correlation

The mutations in the *LMNA* gene associated with skeletal muscle involvement are mostly autosomal dominant. However, a few EDMD cases caused by recessive *LMNA* mutations have been described and defined as EDMD3 [68,69]. These cases were associated with homozygous mutations and displayed usually severe and early EDMD with contractures, whereas cardiac involvement, if any, was only late and mild.

**Figure 2:**
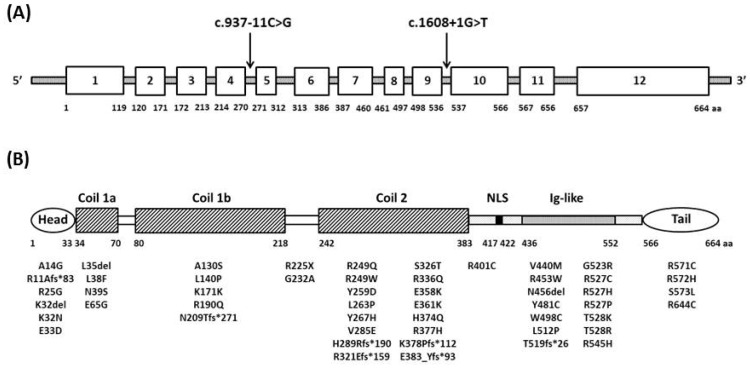
This is a schematic representation of the *LMNA* gene (**A**) and the lamin A/C protein (**B**). Mutations associated with laminopathy and identified in Italian patients are indicated with the corresponding nucleotide (**A**) or amino acid (**B**) changes. The globular head and tail domains are shown as an oval. The coil 1 (segments 1A and 1B), and coil 2 (segments 2A and 2B), constituting the α-helical rod domain, are shown as rectangles. Double lines represent linkers L1, L2 and L12. NLS = nuclear location signal; Ig = immunoglobulin. Modified from Neurology 2014, 83, 1634–1644 [5].

The high clinical variability, also within the same family, associated with *LMNA* mutations makes it challenging to establish genotype-phenotype correlations [5]. In an Italian retrospective study of a cohort of 78 *LMNA*-RD patients, *LMNA* missense mutations were mainly identified in patients with EDMD2, in those affected by *LMNA*-CMD and in patients without cardiac involvement, as already suggested by a previous study [70], whereas frameshift mutations were more frequently detected in LGMD1B patients and in those with heart involvement [5,71]. In EDMD2 and LGMD1B patients, gene variants mainly clustered in the immunoglobulin-like (exon 7–10) and coil 2B (exon 6) regions, respectively, which are regions crucial for the interactions of lamins with the inner nuclear membrane lamin binding proteins and lamin dimerization [2,9]. Mutations associated with *LMNA*-CMD were spread across the N-terminal and the first part of the rod domains (exon 1 and exons 4 and 5); mutations restricted to the tail domain were not significantly associated with heart involvement, suggesting that myocardium might be more sensitive to modifications in the N-terminal portion of lamin A/C than skeletal muscle [71] (Figure 2).

Environmental factors and additional possible genetic modifiers have been postulated to explain the phenotypic variability; in particular, some studies revealed a possible pathogenetic role for genes that can modulate the effect of lamin A/C mutations or a digenic mechanisms, by which mutations in *LMNA* and in another gene may be concomitant in the same patient [72,73,74,75,76,77]. For this purpose, the emerging data on the role of other nuclear envelope components and proteins interacting with A-type lamins in human physiology and pathology may provide further clues to explain the phenotypic variability of skeletal muscle laminopathies.

The vast majority of overlapping syndromes and atypical phenotypes are caused by dominant, in-frame mutations, which are spread throughout the entire *LMNA* gene, but affecting more often the exons 1,2, 8 and 9 [6]. It was supposed that in-frame mutations are capable of interfering with the stability of lamin filaments and also with the numerous A type lamins’ binding partners [78]; thus, the effect of these in-frame mutations could consist of the perturbation of the physiologic processes regulated by lamins at the different tissue levels.

Interestingly, adipose tissue defects and premature aging syndromes, whose mutations are clustered in the N- and C-terminal domains, are each associated with a hotspot mutation, accounting for about 80% of the patients [2]. In addition, non-sense and out-of-frame mutations are extremely rare in these phenotypes and detected almost exclusively in patients with skeletal muscle laminopathies [2].

## 7. Treatment

To date, there is no specific therapy for laminopathies. However, symptomatic treatment in the context of a multidisciplinary medical team has been shown to be helpful for the management of orthopedic and cardiac complications. In this regard, orthopedic surgical and conservative procedures may prevent progression or improve contractures and scoliosis. Implantation of a cardioverter defibrillator has been recommended to prevent sudden death due to lethal tachyarrhythmias [40]; on the contrary, pacemaker implantation does not reduce the risk of sudden death. No definite data are available on the effect of the conventional medications, such as angiotensin-converting enzyme inhibitor on the progression to dilated cardiomyopathy or heart failure in patients mutated in *LMNA.*

No clinical trial is ongoing in patients affected by skeletal muscle laminopathies [79]. However some promising results have been observed in preclinical studies, mainly in progeria, lipodystrophies and dilated cardiomyopathies [25].

Stem cell treatment may represent a therapeutic option. Recently, Catelain *et al.* [80] injected bone morphogenic protein 2-committed embryonic stem cells (ESCs) and myoblasts at four sites on the anterior-lateral wall of the left ventricle of a mouse model of dilated cardiomyopathy caused by *LMNA* mutation (*Lmna*^H222P/H222P^ mouse). After four and eight weeks of transplantation, few or no ESCs were detected in the heart, whereas muscle cells were observed together with a stabilization and improvement of heart function. These findings are promising and pave the way toward new ‘personalized’ therapeutic approaches in laminopathies.

Exon skipping, a therapy restoring the reading frame or switching protein isoforms, is under clinical trial in other diseases (e.g., in patients with Duchenne muscular dystrophy). The rationale is that removing an in-frame exon containing a pathogenic mutation, could improve the phenotype. Antisense oligonucleotides skipping *LMNA* exon 5 in human cells demonstrated the possibility of treating specific laminopathies with this approach [81]. However, given the low frequency of nonsense pathogenic *LMNA* mutations [2,5], this approach may be suitable only for a minority of cases.

Another molecular approach to be applied to laminopathies should be the use of RNA interference (RNAi). This therapy has been tested in Hutchinson-Gilford progeria syndrome (HGPS), in which mutant *LMNA* mRNAs are selectively destroyed using short hairpin RNA (shRNA) or synthetic oligonucleotides with long half-lives. These approaches were shown to effectively restore several cellular and nuclear phenotypes [82,83]. However, systemic delivery of shRNA or synthetic oligonucleotide is a major hurdle for any gene therapy. Progerin is known to accumulate in patient-derived vascular cells from skin [83], and the progressive loss of vascular smooth muscle cells has been hypothesized as the cause of fatal cardiac complications in the HGPS mouse model [84]. A molecular therapy by RNAi able to reduce the accumulation of mutant A-type lamin proteins might be a treatment of choice for those *LMNA* mutations associated with specific phenotypes in which accumulation of the toxic mutant lamin is the predominant pathogenic mechanism.

Another promising therapy is rapamycin, which efficiently and selectively triggers lysosomal degradation of farnesylated prelamin A, the most toxic processing intermediate. Treatment of MAD cells with rapamycin was able to restore SIRT-1 localization and the distribution of chromatin markers, elicits the release of the transcription factor Oct-1 and determines the shortening of the prolonged S-phase [26]. Recently, a combination of all-trans-retinoic acid and rapamycin was shown to significantly improve the cellular phenotype in HGPS [85]. Moreover, the *in vivo* administration of temsirolimus, a rapamycin analog, was able to prevent the deterioration of cardiac function in hearts of mice with cardiomyopathy caused by the *LMNA* mutation and with the AKT-mTOR pathway hyperactivated [86,87]. A defective autophagy in the hearts of these mice was also observed that ameliorated in correlation with the improvement in heart function induced by pharmacological interventions [86], providing a rationale for a novel treatment of *LMNA* cardiomyopathy.

## 8. Conclusions

Encouraging data on *in vitro* and *in vivo* laminopathy models provided clues for possible future treatments in human beings. However, most of the studies concentrated on progeria, lipodystrophies and dilated cardiomyopathy, whereas few data are available for skeletal muscle laminopathies; higher effort is recommended in this regard.

In addition, some issues limit the chance for clinical studies aimed to find efficacious therapies for skeletal muscle laminopathies. First, considerable clinical overlap among the different skeletal muscle laminopathy phenotypes suggests they should be considered as a continuum in the clinical spectrum of *LMNA*-RD and makes it difficult to establish genotype-phenotype correlations and to fully predict their natural history; hence, prospective studies on a large cohort of patients are needed, probably through world-wide national registries, also to clarify the management of cardiac issues in younger patients. Second, our knowledge of the genetic and pathogenic mechanisms of laminopathies is still incomplete, making it challenging to find effective drugs. Lastly, due to the apparent slow progression of muscle weakness and contractures over the years, it is difficult to set up a clinical trial aimed to investigate the drug efficacy in 1–2 years, as conventionally performed in most of the pharmacological studies. For these purposes, further studies are needed to overcome the present limitations and to improve the standard of care in patients affected by skeletal muscle laminopathies.

## Figures and Tables

**Table 1:** Clinical and genetic characteristics of patients with laminopathy.

Onset	EDMD2 2nd–3rd Decade	LGMD1B 3rd–4th Decade	*LMNA*-CMD <2 Years
Weakness distribution	scapulo/humero/peroneal	pelvic/scapular	diffuse or DHS
Tendon contractures	frequent, elbow quite specific	relatively frequent	frequent
Axial involvement	frequent	rare	frequent
Scoliosis	frequent	rare	frequent
Rigid spine	frequent	rare	frequent
Dysphagia	very rare	very rare	rare
Facial weakness	rare	very rare	rare
Non-autonomous ambulation	rare and late	rare and late	frequent
Heart involvement	almost invariably with age	almost invariably with age	relatively frequent
Respiratory involvement	rare	rare	relatively frequent
Muscle biopsy	Unspecific myopathic	unspecific myopathic	myopathic or dystrophic
Type of mutations	missense	frameshift	missense

EDMD2: autosomal dominant Emery-Dreifuss muscular dystrophy; LGMD1B: limb-girdle muscular dystrophy type 1B; *LMNA*-CMD: *LMNA*-congenital muscular dystrophy; DHS: dropped head syndrome.
